# Supplementary material for: Improving fake news classification using dependency grammar
Source: PLoS One. 2021 Sep 14;16(9):e0256940. doi: 10.1371/journal.pone.0256940 (PMC8439466; doi:10.1371/journal.pone.0256940)
Supplement: Attachment — Submitted filename: rebuttal_letter.docx [file pone.0256940.s002.docx]

Kitti Nagy

Department of Informatics

Faculty of Natural Sciences

Constantine the Philosopher University in Nitra

Trieda A. Hlinku 1

94901 Nitra

Slovakia

kitti.nagy@ukf.sk

July 8th, 2021

# **Cover Letter**

Dear Editors, Dear Reviewers,

Thank you for your valuable feedback and contribution to improving the article "Improving Fake News Classification Using Dependency Grammar".

We have improved the formatting of the paper and changed the name of the figures.

We have clearly added the main contribution of the work and improved the paper based on all reviewers’ recommendations.

We believe that the improved manuscript meets the required quality standards and is now suitable for publication in Plos One.

Please, find below our response to the reviewer’s comments.

Kitti Nagy

Corresponding author

On behalf of all authors.

# **The List of Responses to the Reviewers’ Comments**

**Review 1:**

*Thank you for your time, effort, and valuable feedback. We appreciate your contribution to improving the comprehensibility and scientific content of the paper. Please, find our answers to your comments and suggestions below.*

**Introduction needs to explain the main contributions of the work more clearly.**

*The introduction was improved. We explained the main aim of the paper in the Introduction more clearly. [p. 5]*

**The Wide ranges of applications need to be addressed in Introductions.**

*A section, where different principles for detecting fake news are introduced, was moved from the Related Work section to the Introduction. [p. 4]. The section was extended by the applications of methods in other fields. [p. 4]*

**There are some grammatical and editing problems in English. English presentation should be further polished, check plagiarism.**

*We have checked the typos and grammar. Some sentences have been rewritten.*

**The motivation for the present research would be clearer, by providing a more direct link between the importance of choosing your own method.**

*We added a few sentences about the motivation for our experiment to the Introduction [p. 5-6].*

**Can cite the following papers like
1. Vehicle theft identification and intimation using gsm & iot
2. Constraint-based measures for DNA sequence mining using group search optimization algorithm
3. AN ENHANCED ALGORITHM FOR FREQUENT PATTERN MINING FROM BIOLOGICAL SEQUENCES**

*Thank you for recommending these insightful papers. The research carried out in the second and third papers motivated us to create new experiments in our future research. We mentioned the future work at the end of the article with the relevant citations. [p. 29]*

**Review 2:**

*Thank you for your time, effort, and valuable feedback. We appreciate your contribution to improving the comprehensibility and scientific content of the paper. Please, find our answers to your comments and suggestions below.*

**What are the limitations of the existing works that motivated the current research?**

*We added a few sentences about the motivation for our experiment to the Introduction [p. 5-6].*

**List out the main contributions of the current work.**

*The main contribution of the work was added to the Introduction [p. 5] and it is stated also in the conclusion [p. 28].*

**The recent works can be summarized in a table.**

*We added a summarization table of research studies as Table 1 [p. 8].*

**Some of the recent and relevant works such as "An ensemble machine learning approach through effective feature extraction to classify fake news, Propagation of Fake News on Social Media: Challenges and Opportunities, A Trusted Social Network using Hypothetical Mathematical Model and Decision-based Scheme" can be discussed in the paper.**

**Compare the current work with recent state-of-the-art.**

*Suggested papers were incorporated into our research work [p. 7, p. 8, p. 11] and were discussed in the paper [p. 28].*

**Provide a detailed analysis on the results obtained.**

*We used several other classification methods (Logistic Regression, SGD Classifier, Multinomial NB, Bernoulli NB, Decision Tree Classifier, Gradient Boosting Classifier) in our research. For evaluation of our proposed techniques, we chose the Linear SVC method, which of the above methods achieved the worst results to the detriment of the MultipleDgw technique and the Random Forest method with the best results for the presented technique.*

*Two methods were selected due to the clarity of the paper. In our opinion, presenting a more detailed analysis of the selected methods would impair the clarity of the paper.*

**The conclusion should include a brief discussion on the limitations of the current work.**

*We have discussed several considerations about the limitations of our approach. At the same time, the final part was expanded with proposals for our future experiments.*

*Thank you again for the reviewers’ effort and all suggestions.*

*We believe that we have correctly understood the reviewers’ comments and recommendations and included them in this improved version of the manuscript.*

*Simultaneously, we believe, we explained a possible contribution of the paper and improved its readability and comprehensibility.*

*Besides the final decision, we appreciate the reviewers' valuable feedback, which motivated us to research fake news identification further.*

*Best regards*

*Kitti Nagy*

*Corresponding author*
